# Clinical efficacy analysis of tip‑flexible suctioning ureteral access sheath combined with disposable flexible ureteroscope to treat 2–4 cm renal stones

**DOI:** 10.1007/s11255-024-04072-y

**Published:** 2024-05-08

**Authors:** Hua Chen, Jiansheng Xiao, Jiaqi Ge, Tairong Liu

**Affiliations:** Department of Urology, Ganzhou People’s Hospital, Jiangxi Medical College, Nanchang University, Ganzhou, Jiangxi China

**Keywords:** Renal calculi, Retrograde intrarenal surgery, Ureteral access sheath, Disposable flexible ureteroscope

## Abstract

**Purpose:**

This study aims to evaluate the clinical efficacy of using a tip‑flexible suctioning ureteral access sheath (TFS-UAS) in combination with a traditional ureteral access sheath (T-UAS) and a disposable flexible ureteroscope (DFU) for treating large renal stones (2–4 cm in diameter).

**Methods:**

We retrospectively collected clinical data from 238 patients who underwent retrograde intrarenal surgery (RIRS) at Ganzhou People’s Hospital between January 2019 and October 2023. The study included 238 patients who met the inclusion criteria, with 125 in the observation group using TFS-UAS and 113 in the control group using T-UAS. We compared differences in the stone-free rate (SFR), complication rates, surgery duration, and average hospital stay between the two groups.

**Results:**

All 238 surgeries were successfully completed. The stone-free rates for the observation group at the first and thirtieth day post-surgery were 87.20% and 95.20%, respectively, whereas for the control group, the rates were 73.45% and 85.84%, showing statistically significant differences (*P* < 0.05). The overall complication rates were 1.6% for the observation group and 14.16% for the control group, also statistically significant (*P* < 0.001). The surgical times for stone removal were (101.17 ± 25.64) minutes for the observation group and (86.23 ± 20.35) minutes for the control group, with significant differences (*P* < 0.05).

**Conclusion:**

Compared to T-UAS, combining TFS-UAS with DFU for treating renal stones of 2–4 cm diameter, although more time-consuming, resulted in higher SFRs and improved safety.

## Introduction

Kidney stones are among the most common urological diseases, with a significantly rising prevalence globally [1]. Treatment has evolved from traditional open nephrolithotomy to minimally invasive techniques. For stones ≤ 2 cm, the European Association of Urology recommends retrograde intrarenal surgery (RIRS) as the preferred treatment [2–4]. For stones > 2 cm, choices vary between centers, mainly between RIRS and percutaneous nephrolithotomy (PCNL) [5, 6]. However, PCNL requires establishing a tract through the renal parenchyma, which is especially demanding for patients without significant hydronephrosis, as it requires considerable surgical skill and experience due to the dense parenchyma filled with stones. Such cases are prone to complications like severe bleeding and infections, and have longer recovery times [7, 8].

Recent advances in endourology have explored the application of RIRS in treating stones larger than 2 cm. Yet, due to limited stone clearance rates in large burden kidney stones, multiple surgeries might be necessary, increasing the risk of intrarenal pressure and potential complications like renal rupture and severe infections [2, 9, 10]. The advent of a TFS-UAS has expanded the indications for ureteroscopic procedures. Its tip can passively flex with the ureteroscope to reach all calyces, allowing for effective stone fragmentation and direct suction of the irrigation fluid and fragments, achieving better outcomes than T-UAS [11].

Currently, the combination of the TFS-UAS and a DFU is primarily used to treat kidney stones smaller than 2 cm, demonstrating good clinical efficacy [11–13]. Research on using this technology to treat stones with a diameter of 2–4 cm has been relatively limited. Since 2017, Ganzhou People’s Hospital has been implementing RIRS technology and, since 2019, has used the TFS-UAS in combination with DFU for stone fragmentation and clearance, successfully treating renal stones of 2–4 cm with favorable results.

## Materials and methods

### Study population

The study included 238 patients who underwent RIRS and met the inclusion criteria between January 2019 and October 2023. Clinical data and surgical procedures were collected for analysis during the perioperative period. The study included participants with a confirmed diagnosis of kidney stones via imaging, stone diameters of 2–4 cm, and no surgical contraindications. Participants were excluded for uncontrolled urinary tract infections, evident stenosis of the affected-side ureter, severe bleeding disorders, or poor physical conditions that precluded surgery. A total of 238 patients met the eligibility criteria for the study. All patients underwent comprehensive urological CT scans, with additional IVU or CTU exams to accurately assess the ureter, renal pelvis, and infundibulopelvic angle (IPA). Patients were divided into two groups according to the type of ureteroscope sheaths used during surgery. The observation group consisted of 125 patients who underwent lithotripsy and stone extraction using TFS-UAS, while the control group included 113 patients who underwent lithotripsy and stone clearance using T-UAS. Clinical and surgical data from the perioperative period were retrospectively collected and compared between the two groups. This study was approved by the Ethics Committee of Ganzhou People’s Hospital, and informed consent was obtained from all patients, who signed informed consent forms.

### Surgical methods

#### Observation group

**Fig. 1:**
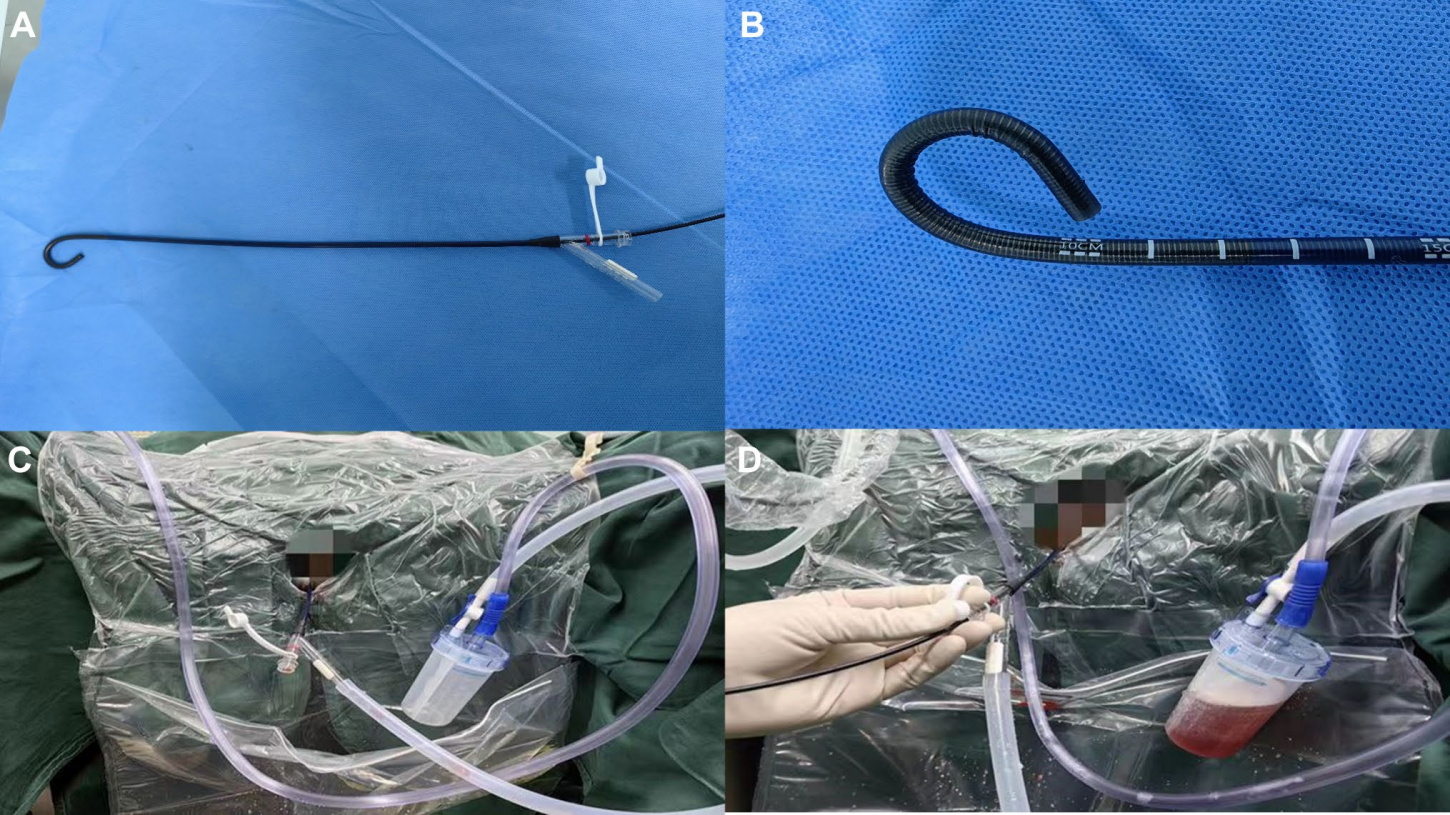
Tip‑flexible suctioning ureteral access sheath and its use during surgery. **A**, **B** Images of the actual tip‑flexible suctioning ureteral access sheath. **C**, **D** Use of the tip‑flexible suctioning ureteral access sheath during surgery

All patients were administered endotracheal anesthesia and positioned for lithotomy. Initial examination with ureteroscope (6/7.5F) confirmed the absence of ureteral stenosis or torsion, allowing for placement of a zebra guidewire upon reaching the renal pelvis and withdrawal of the ureteroscope. A TFS-UAS (12-14F) was inserted along the guidewire, using a 45 cm sheath for males and a 38 cm sheath for females (Fig. 1A, B). The sheath’s suction interface was connected to a vacuum suction bottle and then to the central suction in the operating room. A disposable flexible ureteroscope was used to navigate the upper ureter via the TFS-UAS under direct vision, reaching the renal pelvis. Under ureteroscopic guidance, stones were located, the TFS-UAS was advanced to the stones, and a 200 μm laser fiber was inserted; settings were adjusted to 1.0 J × 30 Hz for continuous stone pulverization. Smaller stones were aspirated through the gap between the sheath and the endoscope, while larger stones were removed by withdrawing the scope (Fig. 1C, D). The renal pelvis and calyces were cleared of stones, after which the integrity of the renal pelvic mucosa was confirmed with no significant bleeding. A guidewire was left in place, the scope was withdrawn, and an F5 double-J stent was inserted along the guidewire, correctly positioned under endoscopic vision and secured with an F5-F6 double-J stent. A urinary catheter was also placed and stones collected in the suction bottle were examined.

#### Control group

Patients were similarly administered endotracheal anesthesia and placed in the lithotomy position. An initial examination with an ureteroscope (6/7.5F) ensured no ureteral stenosis or torsion before placing a zebra guidewire upon reaching the renal pelvis and withdrawing the ureteroscope. A T-UAS (12-14F) was then advanced over the guidewire, with males using a 45 cm sheath and females a 38 cm sheath. A disposable flexible ureteroscope was introduced through the T-UAS to the upper ureter and into the renal pelvis under direct vision. With the aid of the ureteroscope, stones were located and a 200 μm laser fiber was introduced; the energy was set to 1.0 J × 30 Hz for continuous pulverization, with some fragments entering the sheath and larger fragments flushed out by withdrawing the scope. Larger fragments visible in the field were enveloped using a “stone-snatching” technique and expelled into the sheath. The renal pelvis and calyces were inspected for any significant remaining stones and the mucosal integrity of the renal pelvis was confirmed with no significant bleeding observed. A guidewire was left in place, the scope was withdrawn, and an F5 double-J stent was positioned accurately under endoscopic guidance, with an F5 double-J stent secured and a urinary catheter placed.

### Perioperative management

#### Preoperative management

Prior to surgery, all patients underwent a comprehensive assessment including urinary system CT scans, electrocardiograms, and biochemical blood tests to evaluate their health. All patients also completed routine urine tests and midstream urine cultures. According to the “Chinese Guidelines for Diagnosis and Treatment of Urological and Andrological Diseases”, patients with negative urine leukocytes and cultures were administered antimicrobial drugs 30 min before surgery to prevent infection. Patients with positive urine leukocytes but negative cultures were treated with broad-spectrum antibiotics 3 days before surgery. Those with positive urine cultures were treated with antimicrobial drugs for 1 week before surgery and had their urine cultures rechecked.

#### Postoperative management

Postoperatively, routine treatments such as anti-infection measures and fluid replenishment were administered, along with standard blood tests, C-reactive protein (CRP), procalcitonin (PCT), and renal function biochemical assessments. At the first postoperative day, the urinary catheter was removed unless the patient experienced severe symptoms such as fever or gross hematuria, in which case the removal was delayed. Abdominal plain films (kidney–ureter–bladder, KUB) and urinary system CT scans were performed to assess the position of the double-J stent and the SFR. Kidney stones with a diameter less than 2 mm were defined as clinically insignificant and considered cleared [14]. One month postoperatively, a CT re-evaluation was conducted; patients with complete stone clearance had their double-J stents removed, while those with residual stones underwent a secondary stone removal procedure.

### Statistical methods

Statistical analysis was conducted using SPSS 26.0 software. Kolmogorov–Smirnov test was used to assess the normality of all numerical data. Normally distributed continuous data were expressed as mean ± standard deviation (x ± s) and analyzed using the *t* test. For categorical data (%), the *χ*2 test or Fisher’s exact test was applied. *P* value < 0.05 was considered as indicating a statistically significant difference between the two groups.

## Results

### Patient and characteristics of kidney stones

The study involved 238 eligible patients, including 141 males and 97 females, aged 27–82 years, with a mean age of 45.5 ± 12.3 years. The observation group included 125 patients who underwent TFS-UAS and the control group included 113 patients who underwent T-UAS. There were no significant differences between the two groups in terms of mean age, gender, body mass index, stone location, and stone diameter (*P* > 0.05), confirming comparability (Table 1).

**Table 1:** Patient characteristics and kidney stone details

Characteristics	Observation group (*n* = 125)	Control group (*n* = 113)	*t*/*χ*^2^	*P* value
Gender				
Male	76	65	2.246	0.134
Female	49	48		
Age (years)	45.62 ± 12.93	46.35 ± 14.88	0.405	0.686
Kidney stone diameter (cm)	2.81 ± 1.56	2.68 ± 1.42	0.670	0.504
BMI (Kg/M^2^)	25.21 ± 3.77	25.66 ± 4.35	0.858	0.394
Kidney stone location				
Left	69	59	0.078	0.780
Right	56	54		
Kidney stone location				
Upper calyx	43	31	1.517	0.468
Middle calyx, renal pelvis	67	69		
Lower calyx	15	13		
Urinary leukocytes				
Negative	29	27	0.016	0.900
Positive	96	86		
Urine culture				
Negative	92	91	1.605	0.205
Positive	33	22		

### Comparison of perioperative and intraoperative data

The observation group had a statistically significant longer average stone extraction time (101.17 ± 25.64 min) compared to the control group (86.23 ± 20.35 min) (*P* < 0.05). The duration of postoperative hospital stay in the observation group (1.53 ± 0.52 days) was not significantly different from that of the conventional ureteroscopy group (1.62 ± 0.63 days). Pre-discharge re-evaluations using KUB and CT scans revealed that the SFR in the observation group was 87.20%, significantly higher than the 73.45% in the control group, with statistically significant differences (*P* < 0.05). Follow-up examinations 30 day post-discharge showed an SFR of 95.20% in the observation group, also exceeding the 85.54% in the control group, with statistically significant differences (*P* < 0.05) (Table 2).

**Table 2:** Comparison of perioperative and intraoperative data

Characteristics	Observation group (*n* = 125)	Control group (*n* = 113)	*t*/*χ*^2^	*P*
Surgical time (min)	101.17 ± 25.64	86.23 ± 20.35	5.001	< 0.001
SFR at postoperative day 1 (%)	109 (87.20)	83 (73.45)	7.195	0.007
SFR at postoperative day 30 (%)	119 (95.20)	97 (85.84)	6.197	0.013
Postoperative hospital stay (days)	1.53 ± 0.52	1.62 ± 0.63	1.206	0.229

### Comparison of complication occurrences

In the comparison of complications, the observation group had one patient with fever and one with renal pelvis mucosal injury during sheath placement. In the control group, six patients developed postoperative fever, five sustained injuries to the renal pelvis or calyceal mucosa, one experienced intraoperative bleeding leading to termination of the procedure, and four developed ureteral “stone streets” which were cleared after a secondary surgical intervention. Neither group suffered from severe complications such as damage to surrounding organs, septic shock, or ureteral rupture. The overall incidence of complications significantly differed between the groups (*P* < 0.001) (Table 3).

**Table 3:** Comparison of complication occurrences

Characteristics	Observation group (*n* = 125)	Control group (*n* = 113)	*χ*2	*P*
Overall occurrence rate of complications	2	16	11.654	< 0.001
Fever (> 38 °C)	1	6	2.794	0.096
Intraoperative bleeding	0	1	0.003	0.960
Mucosal injury	1	5	1.870	0.172
Steinstrasse	0	4	2.613	0.106

## Discussion

In China, the prevalence of kidney stones is approximately 6%, with a higher incidence rate in the southern region compared to the northern region [15]. Kidney stones can cause recurring symptoms such as pain, hematuria, and urinary frequency/urgency. Large stone burdens may result in severe complications, including uremic syndrome, chronic kidney disease, urinary perforations, and renal effusion [16, 17]. Recent advancements in flexible ureteroscope manufacturing, endoscopic surgical techniques, and stone fragmentation tools like holmium lasers have made RIRS the preferred technique for managing small-burden renal stones. T-UAS plays a crucial role in RIRS, although it is associated with high rates of residual stone fragments [18, 19]. Studies indicate that residual fragment rates for sizes smaller than 3 mm, 2 mm, and 1 mm are 16.7%, 48.5%, and 77.8%, respectively [20]. Moreover, prolonged use of T-UAS in lithotripsy, lacking negative pressure suction or pressure control, can increase the risk of complications like bleeding and infections. Research has shown that the single-session stone clearance rate for ureteroscopic lithotripsy exceeds 90% for renal or upper ureteral stones smaller than 2 cm [21]. Conventional ureteroscopy often does not achieve complete stone removal in a single session for large stones (> 2 cm) and may lead to the formation of steinstrasse within the ureter, necessitating multiple surgeries [22]. Furthermore, studies reveal that the single-session stone clearance rate decreases with increasing stone size, dropping to as low as 62% [23]. In addition, prolonged operative times are linked to an increased risk of complications such as bleeding and infection [24–27].

To minimize surgical complications, some studies have utilized modified ureteroscopes with suction sheaths. However, the distal end of these sheaths reaches only the pelviureteric junction and cannot extend into the renal pelvis or calyces, resulting in low stone retrieval efficiency [25]. In addition, the UAS opening is prone to obstruction, which elevates renal pelvis pressure and causes reflux, increasing the risk of bleeding and infection [28]. Previous studies have shown that TFS-UAS reduces intrarenal pressure (IRP), increases SFR, shortens operative times, and lowers complication rates [29, 30]. TFS-UAS negative pressure suction sheaths, compared to T-UAS, provide flexible access to calyceal stones. They allow the sheath’s front end to be positioned precisely within a calyx for effective stone fragmentation and retrieval, aiming to reduce residual stones and recurrence [25, 31]. The simultaneous suction feature aids in stone aspiration and importantly, facilitates the rapid removal of irrigation fluids during surgery. This maintains a low-pressure environment in the renal pelvis, helping to prevent severe intraoperative and postoperative infections [27].

In this study, we used TFS-UAS combined with DFU to manage large renal calculi with diameters ranging from 2 to 4 cm, achieving relatively positive outcomes. This study observed that the SFR on the first and thirtieth postoperative days using TFS-UAS were 87.20% and 95.20%, respectively, significantly higher than the control group’s rates of 73.45% and 85.84%. The observation group experienced complications including one case of postoperative fever and another of mucosal damage in the renal pelvis during sheath placement, with an overall complication rate of only 1.6%, which was lower than the control group’s rate of 14.15%.

First, the TFS-UAS features a wide range of bending angles, allowing access to the renal pelvis, upper-middle, and most lower calyces, while preserving the ureteroscope’s bending and steering flexibility. Consequently, flexible control of the aspiration sheath during surgery leads to high stone clearance rates and reduced complications. Second, the technique uses low negative pressure aspiration to maintain a low-pressure state in the renal pelvis, drawing perfusion fluid into the aspiration bottle while keeping the renal pelvis or calyces adequately filled. The combination of these factors facilitates flexible movement of the ureteroscope within the renal pelvis and calyces. This approach allows for the effective removal of stone debris via efficient irrigation fluid circulation, maintains clear visibility, and prevents laser energy-induced damage to the renal pelvis and calyceal mucosa. In patients with infectious stones, the renal pelvic mucosa becomes more fragile from prolonged infection and stone irritation, elevating the risk of bleeding. In such cases, stones are fragmented before retrieval without aspiration during the fragmentation process to mitigate this risk. Stone aspiration uses lower suction levels to avoid mucosal damage from excessive aspiration force, thus preventing renal pelvic mucosal bleeding during surgery. However, clearing kidney stones from lower calyces with challenging angles continues to be difficult. To address these stones, the study positions the sheath opening as close to the calyceal neck as possible when the IPA is too acute for direct access. The ureteroscope is inserted into the calyx, and the assistant is instructed to apply pressure on the affected renal area to minimize angles and facilitate stone location. Irrigation flow is moderately increased, using high-pressure irrigant to help flush out fragmented stones.

Unlike other studies [12, 13], the average operative time for the observation group in this study was notably longer compared to the control group. In the observation group, the flexible ends of the ureteroscopic sheaths could reach all renal calyces, allowing for the effective use of this flexibility during surgery to extract shattered stones via vacuum suction. However, larger stone fragments (approximately 2–3 mm in diameter) were challenging to remove within the confined space of the sheath, necessitating the withdrawal of the ureteroscope to facilitate stone extraction. To maximize the stone-free rate in a single procedure, frequent scope withdrawals for stone clearance were required, consuming additional surgical time. In contrast, the control group employed a method aimed at pulverizing stones, as their sheaths could not reach the renal calyces, leaving more fragments to be expelled postoperatively via a double-J stent. Consequently, the average surgical time for the observation group was (101.17 ± 25.64) minutes, longer than that for the control group (86.23 ± 20.35) minutes. However, the observation group, equipped with vacuum suction control, maintained a clear surgical field and safe intrapelvic pressures, hence not increasing the complication rate with extended surgery time unlike traditional ureteroscopic sheaths. Initially, using flexible sheaths, the technique of simultaneous fragmentation and suction occasionally caused larger particles to lodge in the sheath gap, potentially damaging the sheath’s plastic surface upon forced removal, yet not impairing its visibility or flexibility. With advancements and improvements in surgical techniques, especially using pulverization to reduce stone size followed by collective suction, the likelihood of ureteroscope damage significantly decreased.

This study has certain limitations; primarily, it utilized a retrospective research methodology and involved a small sample size, which may affect the accuracy of the conclusions. Initially, standard ureteroscopic sheaths were used for stone removal, while later stages predominantly used flexible-tip suction sheaths, and the chronological variation might also influence the outcomes. Therefore, a larger-scale prospective comparative study is required to validate our hypotheses.

## Conclusion

In conclusion, the use of TFS-UAS combined with DFU for treating renal stones of 2–4 cm in diameter demonstrates a high stone clearance rate and good safety profile, indicating substantial clinical value.

## Data Availability

The raw data supporting the conclusions of this article will be made available by the authors, without undue reservation.
